# Patterns of Left Ventricular Remodelling in Children and Young Patients with Hypertrophic Cardiomyopathy

**DOI:** 10.3390/jcm13133937

**Published:** 2024-07-04

**Authors:** Emanuele Monda, Martina Caiazza, Chiara Cirillo, Marta Rubino, Federica Verrillo, Giuseppe Palmiero, Gaetano Diana, Annapaola Cirillo, Adelaide Fusco, Natale Guarnaccia, Pietro Buono, Giulia Frisso, Paolo Calabrò, Maria Giovanna Russo, Giuseppe Limongelli

**Affiliations:** 1 Department of Translational Medical Sciences, Inherited and Rare Cardiovascular Diseases, University of Campania “Luigi Vanvitelli”, 80131 Naples, Italy; martina.caiazza@yahoo.it (M.C.); kiaracirillo@gmail.com (C.C.); rubinomarta@libero.it (M.R.); fedeverrillo@gmail.com (F.V.); g.palmiero@hotmail.it (G.P.); gaetanodiana1991@gmail.com (G.D.); cirilloannapaola@gmail.com (A.C.); adelaidefusco@hotmail.it (A.F.); natale.0196@gmail.com (N.G.); paolo.calabro@unicampania.it (P.C.); mariagiovanna.russo@unicampania.it (M.G.R.); limongelligiusepppe@libero.it (G.L.); 2 Directorate General of Health, Campania Region, 80131 Naples, Italy; pietro.buono2@regione.campania.it; 3 Department of Molecular Medicine and Medical Biotechnology, University of Naples Federico II, 80131 Naples, Italy; gfrisso@unina.it

**Keywords:** hypertrophic cardiomyopathy, left ventricular hypertrophy, remodelling

## Abstract

**Introduction**: The aim of this study was to evaluate the age at onset, clinical course, and patterns of left ventricular (LV) remodelling during follow-up in children and young patients with hypertrophic cardiomyopathy (HCM). **Methods**: We included consecutive patients with sarcomeric or non-syndromic HCM below 18 years old. Three pre-specified patterns of LV remodelling were assessed: maximal LV wall thickness (MLVWT) thickening; MLVWT thinning with preserved LV ejection fraction; and MLVWT thinning with progressive reduction in LV ejection fraction (hypokinetic end-stage evolution). **Results**: Fifty-three patients with sarcomeric/non-syndromic HCM (mean age 9.4 ± 5.5 years, 68% male) fulfilled the inclusion criteria. In total, 32 patients (60%) showed LV remodelling: 3 patients (6%) exhibited MLVWT thinning; 16 patients (30%) showed MLVWT thickening; and 13 patients (24%) progressed to hypokinetic end-stage HCM. Twenty-one patients (40%) had no LV remodelling during follow-up. In multivariate analysis, MLVWT was a predictor of the hypokinetic end-stage remodelling pattern during follow-up (OR 1.17 [95%CI 1.01–1.36] per 1 mm increase, *p*-value 0.043), regardless of sarcomeric variants and New York Heart Association class. Two patients with sarcomeric HCM, showing a pattern of MLVWT regression during childhood, experienced progression during adolescence. **Conclusions**: Different patterns of LV remodelling were observed in a cohort of children with sarcomeric/non-syndromic HCM. Interestingly, a pattern of progressive MLVWT thinning during childhood, with new progression of MLVWT during adolescence, was noted. A better understanding of the remodelling mechanisms in children with sarcomeric HCM may be relevant to defining the timing and possible efficacy of new targeted therapies in the preclinical stage of the disease.

## 1. Introduction

Hypertrophic cardiomyopathy (HCM) (OMIM #192600) is defined as a myocardial disease characterised by left ventricular (LV) hypertrophy not solely explained by hemodynamic overload [1,2]. Sarcomeric gene protein disease represents the most common cause of HCM, both in children and adults [1]. The identification of a sarcomeric gene pathogenic variant is observed in up to 60% of cases, with *MYH7* and *MYBPC3* representing the most commonly affected genes. However, in some cases (up to 5–10%), non-sarcomeric causes of HCM can be observed, including syndromic conditions, glycogen storage disorders, and mitochondrial and neuromuscular diseases [3]. The overall prevalence of HCM in children is estimated to be 0.002–0.005% [4].

Several myocardial diseases exhibit LV remodelling during follow-up, consisting of various changes in ventricular architecture, such as dilation of the LV cavity or myocardial thinning or thickening [5]. The pattern of LV remodelling has a significant impact on the disease course, potentially affecting the natural history and management of the condition.

While different LV remodelling patterns have been described in adults with sarcomeric HCM or in children with specific causes of HCM (e.g., Noonan syndrome with multiple lentigines, infants of diabetic mothers) [6,7,8,9], the clinical progression of LV hypertrophy in patients with sarcomeric or non-syndromic HCM during childhood or adolescence is poorly characterised. A better understanding of the natural disease course may help clinicians in improving risk stratification, predicting adverse outcomes, and personalising management, including the future timing of the introduction of disease-modifying therapies.

The aim of this study was to evaluate the age at onset, clinical course, and patterns of LV remodelling in children and young patients with sarcomeric or non-syndromic HCM.

## 2. Methods

A retrospective, longitudinal single-centre cohort of consecutive children (<18 years old) diagnosed with HCM between 2002 and 2018 was established. Clinical follow-ups were available up to December 2020. The study was conducted at a high-volume HCM centre: the Inherited and Rare Cardiovascular Diseases Unit, Department of Translational Medical Sciences, University of Campania “Luigi Vanvitelli”, Monaldi Hospital, Naples, Italy. Approval from the Internal Review Board Committee was obtained, and the study complies with the principles of Good Clinical Practice and the Declaration of Helsinki.

### 2.1. Eligibility Criteria

Patients diagnosed with sarcomeric or non-syndromic HCM under the age of 18 were eligible for inclusion. The diagnosis of HCM was confirmed if the LV wall thickness exceeded two standard deviations above the body surface area-corrected population mean (z-score ≥ 2), a finding not solely attributable to abnormal loading conditions [1,10]. Patients were classified as having sarcomeric HCM if they exhibited a pathogenic or likely pathogenic (P/LP) variant in one of the eight core sarcomeric genes: myosin-binding protein C (*MYBPC3*), myosin heavy chain (*MYH7*), cardiac troponin T (*TNNT2*), cardiac troponin I (*TNNI3*), a-tropomyosin (*TPM1*), myosin essential and regulatory light chains (*MLY2*, *MYL3*), and actin (*ACTC*). Patients were classified as having non-syndromic HCM if genetic testing did not identify a P/LP variant in a sarcomeric gene or if they did not undergo genetic testing, but other genetic syndromes, metabolic disorders, neuromuscular disorders, and congenital heart diseases (e.g., subaortic valve stenosis) were clinically excluded [11].

### 2.2. Data Collection

Eligible patients were identified by the principal investigator using multiple sources, including medical records and medical databases. The search strategy involved the use of keywords such as “hypertrophic cardiomyopathy” and “LV hypertrophy”. All cases diagnosed as HCM under the age of 18 were retrieved and then assessed for the inclusion and exclusion criteria. Anonymised clinical data were collected, encompassing non-identifiable demographics, family history, symptoms, resting ECG, 2D, Doppler, and colour transthoracic echocardiography, as well as genetic testing results. Clinical evaluations at our centre, including standard ECG and echocardiography, were conducted every 6 months. Data were collected from the baseline assessment to the last clinical review.

### 2.3. Clinical Investigations

Echocardiographic measurements were performed according to current guidelines [12]. Specifically, end-diastolic LV wall thickness was measured by 2D echocardiography in the parasternal short-axis view in four places at the level of the mitral valve and papillary muscles (anterior and posterior septum, lateral and posterior wall), and in two places at the apical level (anterior and posterior septum). Maximal LV wall thickness (MLVWT) was defined as the greatest thickness in any single segment [1].

### 2.4. Genetic Testing and Variant Classification

Patients underwent genetic analysis using a next-generation sequencing (NGS) panel containing 202 genes, including both sarcomeric and non-sarcomeric genes (e.g., RAS-MAPK genes, metabolic genes). Extensive details of the NGS panel and procedure have been previously described [13]. Genetic testing was performed after obtaining informed written consent.

### 2.5. Left Ventricular Remodelling

We defined a priori three different patterns of LV remodelling during follow-up: 1. an increase ≥15% in the MLVWT in both mm and z-score (MLVWT thickening); 2. a reduction ≥15% in the MLVWT in both mm and z-score (MLVWT thinning) not associated with a reduction in LV ejection fraction; and 3. a reduction of ≥15% in the MLVWT in both mm and z-score associated with a reduction in LV ejection fraction (hypokinetic end-stage evolution). A z-score that remained stable during follow-up was defined as having no LV remodelling or stability. The LV remodelling pattern was evaluated throughout the entire follow-up period and only in patients with ≥12 months of follow-up. We chose 15% as the cut-off to define MLVWT thinning or thickening in order to minimise potential bias related to inter- and intra-observer variability in echocardiographic LV measurements [5,14].

### 2.6. Statistical Analysis

Body surface area (BSA) was calculated from height and weight [15]. The 2D MLVWT is expressed in millimetres and z-scores corrected for BSA, using normative data validated in a large cohort of healthy individuals (http://www.parameterz.com/refs/lopez-circimaging-2017, accessed on 1 March 2024) [16]. MLVWT z-scores were recalculated retrospectively to give uniformity among patients evaluated in different eras. Normally distributed continuous variables are described as mean ± standard deviation, with two- or three-group comparisons conducted using Student’s t-test and analysis of variance (ANOVA), respectively. Skewed data are described as median (interquartile range [IQR]), with two- or three-group comparisons performed using Wilcoxon rank-sum and Kruskal–Wallis tests, respectively. Categorical variables are listed as numbers (percentage), with group comparisons conducted using a χ^2^ test or Fisher’s exact test. A significance level (*p*-value) of 0.05 (two-sided test) was used for all the comparisons. Univariate analysis of clinically relevant characteristics was performed. A stepwise regression, which included measures with a univariate *p*-value of ≤0.05, was used to build the multivariate model. Results are presented as odds ratios (ORs), 95% confidence intervals (CIs), and 2-sided *p*-values. All statistical analyses were performed using IBM SPSS Statistics for Macintosh, Version 27.0 (IBM Corp., Armonk, NY, USA).

## 3. Results

### 3.1. Enrolment and Baseline Characteristics

Among the 60 patients with a diagnosis of HCM at <18 years old, who were retrospectively identified, we excluded 7 patients with missing data at baseline evaluation or follow-up. The remaining 53 patients represent the final study cohort.

The clinical characteristics of the study population are shown in Table 1. The age at baseline evaluation was 8.8 ± 5.5 years, and 36 patients (68%) were male. A family history of HCM was present in 26 patients (49%), and a family history of sudden cardiac death (SCD) was present in 18 patients (34%). Among the 53 patients included in the study, 38 (72%) underwent genetic testing and 23 (44%) showed a P/LP variant in sarcomeric genes: 6 patients (11%) had a P/LP in *MYBPC3*, 12 (23%) in *MYH7*, 3 (6%) in *TNNT2*, and 2 (4%) in *TPM1* (Table 2). ECG abnormalities were observed in 45 patients (85%). A review of the baseline echocardiograms demonstrated an average MLVWT of 16.9 ± 5.9 mm (z-score 9.1 ± 4.7). LV outflow tract obstruction was observed in 18 patients (33.9%) and required surgical myectomy in 3 cases.

### 3.2. Left Ventricular Remodelling

**Figure 1:**
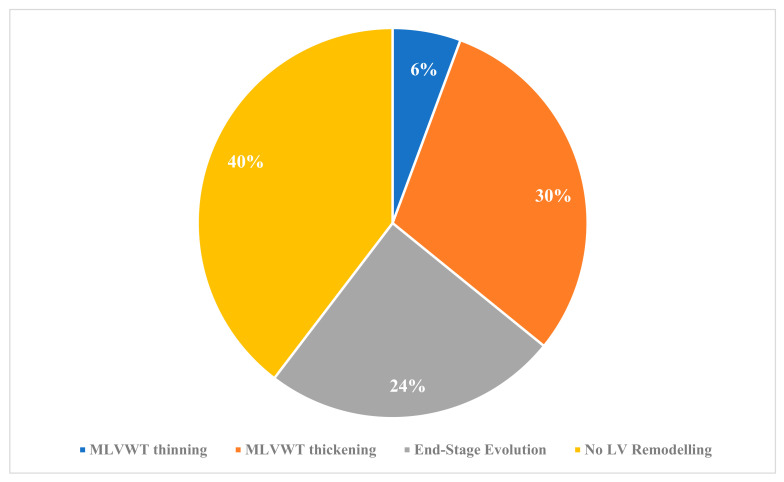
Patterns of left ventricular remodelling during follow-up. Abbreviations: LV, left ventricular; MLVWT, maximal left ventricular wall thickness.

**Figure 2:**
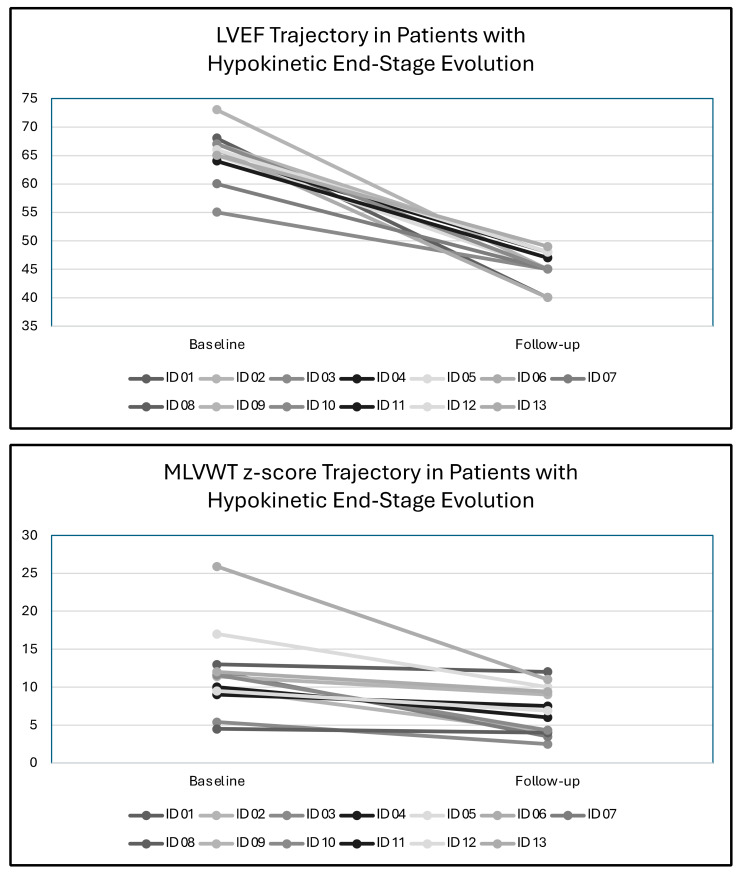
Left ventricular ejection fraction and maximal left ventricular wall thickness trajectories in patients who develop hypokinetic end-stage remodelling. Abbreviations: LVEF, left ventricular ejection fraction; MLVWT, maximal left ventricular wall thickness.

The different patterns of LV remodelling during follow-up are shown in Figure 1. Over a median follow-up of 9.4 ± 4.7 years, MLVWT thickening was the most common type of LV remodelling (*n* = 16, 30%), followed by hypokinetic end-stage evolution (*n* = 13, 24%) and MLVWT thinning (*n* = 3, 6%). No LV remodelling was observed in 21 patients (40%). Among these 21 patients, although there was no significant reduction in the absolute value of MLVWT, 10 patients exhibited a significant reduction in MLWVT z-score due to the increase in BSA during growth. In the three patients showing MLVWT thinning, the reduction in MLVWT was associated with an increase in LV end-diastolic diameter and no reduction in LVEF. In contrast, patients showing hypokinetic end-stage evolution showed a reduction in MLVWT and in LVEF (Figure 2).

### 3.3. Left Ventricular Remodelling According to Age at Presentation

Regarding diagnosis, 8 patients (15%) were diagnosed in infancy, 21 (39%) in childhood, and 24 (45%) in adolescence. Patients presenting in childhood or adolescence more commonly had a higher NYHA class and less commonly experienced MLVWT thinning during follow-up compared to those presenting in infancy. No significant differences in other clinical, genetic, and echocardiographic parameters were observed between the three groups (Table 3).

Two patients with sarcomeric HCM showed two different patterns of LV remodelling during follow-up. In the first patient, HCM was diagnosed at 1 month, with detection of MLVWT at the anterior septum equal to 12 mm (z-score 11.5). Septal thickness remained stable during follow-up (11.5 mm at 9 years old, z-score 2.8). However, at the last examination (16 years old), the patient showed an MLVWT of 26 mm (z-score 5.9). Patient 2 showed a similar course: HCM was diagnosed at 8 months (MLVWT 12 mm, z-score 5.6), and septal thickness remained stable during follow-up (12 mm at 14 years old, z-score 2.8) and showed progression during adolescence (18 mm at 17 years old, z-score +7.6).

### 3.4. Risk Factors for LV Remodelling during Follow-Up

Patients experiencing MLVWT thinning during follow-up were younger compared to those with other LV remodelling patterns or no LV remodelling. In contrast, patients with hypokinetic end-stage evolution had higher MLVWT at baseline evaluation than patients with other LV remodelling patterns or no remodelling (Table 4).

At univariate analysis, P/LP variants in *MYH7* (OR 5.88 [95%CI 1.30–26.51], *p*-value 0.021), NYHA class II (OR 6.00 [95%CI 1.42–25.27], *p*-value 0.015), and MLVWT (OR 1.22 [95%CI 1.07–1.40] per 1 mm increase, *p*-value 0.003) were predictors of the hypokinetic end-stage remodelling pattern during follow-up. However, when accounting for *MYH7* mutation status and NYHA class, the only risk factor maintaining its independent predictive value was MLVWT (OR 1.17 [95%CI 1.01–1.36] per 1 mm increase, *p*-value 0.043) (Table 5).

## 4. Discussion

HCM is a heterogeneous disease related to pathogenic variants in the sarcomeric gene in approximately 60% of patients [1,10,17]. Of clinical interest, the genetic background of HCM overlaps with other cardiomyopathies, such as restrictive and dilated cardiomyopathies [18].

Traditionally viewed as a disease of adolescents and adults, sarcomeric gene defects have also been reported in infants and children with HCM [19]. The natural history and clinical progression of LV hypertrophy have been extensively studied in adults, and the progression of LV hypertrophy in adults with HCM is the most common pattern of LV remodelling [5]. Additionally, the thinning of the myocardial wall associated with progressive systolic dysfunction, known as hypokinetic or end-stage HCM, is a well-documented phenomenon in a subgroup of patients [20], particularly in adults with unfavourable genotypes such as double or compound mutations, but it is less frequent in children, except in those forms associated with metabolic or mitochondrial disorders [19]. Recently, a favourable pattern of LV thinning, not associated with a reduction in LV ejection fraction and worse outcome, has been reported in adults with HCM [5]. However, the clinical course of LV hypertrophy in childhood HCM remains poorly characterised.

The main findings of this study were as follows: LV remodelling was observed in 60% of the patients, with MLVWT thickening representing the most common form; hypokinetic end-stage evolution was common, with MLVWT at baseline representing the most important risk factors at multivariate analysis; a specific pattern of remodelling was observed in two patients, with MLVWT regression during childhood and new progression during adolescence; and patients experiencing MLVWT thinning during follow-up were younger compared to patients presenting other LV remodelling patterns or no LV remodelling.

In our cohort of 53 patients with sarcomeric HCM, 30% showed a progression of MLVWT, 24% showed a hypokinetic end-stage evolution, and 6% showed LV thinning with preserved LV ejection fraction. These patterns have been already described in adults with HCM [5]. However, we observed a relatively high number of patients with end-stage evolution (24%). This is an important finding, since end-stage evolution is considered to be typically associated with mitochondrial and metabolic disorders [21]. This phenotype, particularly during adolescence, seems to be more frequent in patients with massive hypertrophy. Previous studies in young adults showed that HCM patients with massive LV hypertrophy had progressive LV wall thinning during follow-up, and this may predispose them to hypokinetic end-stage evolution, particularly in young patients [22,23,24]. This seems consistent with our finding, showing an association between massive LV hypertrophy and wall thinning/end-stage evolution. We also noted an apparently favourable evolution (LV wall thinning) in a small subgroup of patients with sarcomeric/non-syndromic HCM with diagnosis in infancy. Whether this is a real phenomenon or related to the lack of large normative data in this population is not known at this stage. In addition, long-term prospective observations are required to understand the clinical significance of these findings.

Moreover, in two patients with sarcomeric HCM, after a relative stabilisation during childhood, we noted a progression of LV hypertrophy during adolescence. This suggests another potentially important clinical implication of the study. It is intuitive that, after foetal life, adolescence represents another “hot phase” for cardiac remodelling. Patients with sarcomeric gene defects may be particularly sensitive to hormonal (e.g., IGF-1, GH) and environmental (e.g., physical activity) factors, which may trigger epigenetic modifications and plastic myocyte modifications [25]. In this sense, patients with an early clinical diagnosis of HCM and subsequent stabilisation/regression of LV hypertrophy during follow-up should be re-evaluated during adolescence, particularly if they carry a disease-causing sarcomeric gene pathogenic variant [25].

### 4.1. Clinical Implications

The results of our findings, along with experimental studies in the literature, indicate that the pathology of HCM may arise very early, even during cardiac development, and imply that the timing and target for future etiological therapies should be reconsidered [26]. Considering the potential impact of complex genotypes in different remodelling pattern evolution, an early clinical and genetic evaluation should be performed.

Sarcomeric mutations typically increase myofilament Ca^2+^ sensitivity and force generation in patients with HCM [27]. In mouse models with cardiomyopathies, the sarcomeric tension developed determines the type of remodelling (concentric in HCM versus eccentric in dilated cardiomyopathy) through differential down-stream activation of the mitogen-activated protein (MAP) kinase ERK1/2 [28]. Mavacamten, a small molecule inhibitor that reduces ATPase and force generation, is capable of blocking or reversing LV hypertrophy, fibrosis, and maladaptive genomic remodelling in an animal model of HCM and has been shown to improve LV outflow tract obstruction and clinical status in adults with HCM [29,30,31,32]. Hence, used at an early stage, it may be of potential therapeutic interest in children with HCM [33]. However, to date, there is a lack of data to support the safety and/or eligibility of this type of medication for the non-adult population.

### 4.2. Study Limitations

This study is limited by the small sample size and has limitations inherent to any retrospective analysis, which include missing data and survival bias. Moreover, genetic analysis was not performed for the entire cohort. Larger studies are needed to better characterise the clinical course of LV hypertrophy in infants and children with HCM and to better understand the possible clinical implications.

## 5. Conclusions

Different patterns of LV remodelling during growth were observed in a cohort of children with sarcomeric/non-syndromic HCM, including a relatively high number of HCM patients with hypokinetic end-stage evolution. Interestingly, a pattern of progressive MLVWT thinning/normalisation during childhood, with a new progression of MLVWT during adolescence, has been noted. A better knowledge of the remodelling mechanisms in children with sarcomeric HCM may be relevant to defining the timing and possible efficacy of new targeted therapies in the preclinical stage of the disease.

## Figures and Tables

**Table 1:** Clinical characteristics of the study cohort. Data are presented as mean ± SD, median (IQR), or *n* (%).

Clinical Features	Study Cohort (*n* = 53)
Age at Baseline, years	8.8 ± 5.5
Male	36 (67.9)
Family History of HCM	26 (49.1)
Family History of SCD	18 (34.0)
Tested for Sarcomeric Variants	38 (71.7)
P/LP Variants in Sarcomeric Genes	
*MYBPC3*	6 (11.3)
*MYH7*	12 (22.6)
*TNNT2*	3 (5.6)
*TPM1*	2 (3.8)
Sarcomeric Negative	15 (28.3)
Double P/LP Variants in Sarcomeric Genes	12 (22.6)
NYHA Class	
I	42 (79.2)
II	11 (20.8)
Abnormal ECG	45 (84.9)
MLVWT, mm	16.9 ± 5.9
MLVWT, z-score	9.1 ± 4.7
LVEF, %	65.7 ± 10.1
Follow-Up	9.4 ± 4.7
Hypokinetic End-Stage Evolution	13 (24.5)
MLVWT Thickening	16 (30.2)
MLVWT Thinning	3 (5.7)
No LV Remodelling	21 (39.6)

Abbreviations: ECG, electrocardiography; HCM, hypertrophic cardiomyopathy; LV, left ventricular; LVEF, left ventricular ejection fraction; MLVWT, maximal left ventricular wall thickness; NYHA, New York Heart Association; P/LP, pathogenic/likely pathogenic; SCD, sudden cardiac death.

**Table 2:** Sarcomeric gene variants identified in the study population.

Variant	Number of Patients
*MYPBC3:* c.1112C>G (p.Pro371Arg)	1
*MYBPC3:* c.2717T>G (p.Val906Gly)	1
*MYBPC3:* c.1483C>G (p.Arg495Gly)	1
*MYBPC3:* c.2306-2A>G	1
*MYBPC3:* c.1855G>A (p.Glu619Lys)	1
*MYBPC3*: c.927-9G>A	1
*MYH7:* c.2155C>T (p.Arg719Trp)	1
*MYH7:* c.2146G>C (p.Gly716Arg)	1
*MYH7:* c.1615A>T (p.Met539Leu)	3
*MYH7:* c.999+55C>G	1
*MYH7:* c.4182C>T (p.Ala1394=)	1
*MYH7:* c.2155C>T (p.Arg719Trp)	2
*MYH7:* c.4954G>T (p.Asp1652Tyr)	1
*MYH7:* c.1357C>T (p.Arg453Cys)	2
*TNNT2:* c.320A>T (p.Lys107Met)	2
*TNNT2:* c.283G>A (p.Val95Met)	1
*TPM1:* c.523G>A (p.Asp175Asn)	1
*TPM1:* c.172G>C (p.Asp58His)	1

**Table 3:** Clinical characteristics of the study cohort according to age at presentation. Data are presented as mean ± SD, median (IQR), or *n* (%).

Clinical Features	Infants (*n* = 8)	Children (*n* = 21)	Adolescents (*n* = 24)	*p*-Value
Age at Baseline, years	0.2 ± 0.1	6.7 ± 3.0	13.6 ± 2.2	<0.001
Males	4 (50.0)	17 (80.9)	15 (62.5)	0.208
Family History of HCM	5 (62.5)	10 (47.6)	11 (45.8)	0.706
Family History of SCD	3 (37.5)	3 (14.3)	12 (50.0)	0.040
Tested for Sarcomeric Variants	6 (75.0)	14 (66.7)	18 (75.0)	0.805
P/LP Variants in Sarcomeric Genes				
*MYBPC3*	1 (12.5)	2 (9.5)	3 (12.5)	0.981
*MYH7*	1 (12.5)	5 (23.8)	6 (25.0)	0.686
*TNNT2*	0 (0.0)	1 (4.8)	2 (8.3)	0.677
*TPM1*	0 (0.0)	1 (4.8)	1 (4.2)	0.804
Sarcomeric Negative	4 (50.0)	5 (23.8)	6 (25.0)	0.329
Double P/LP Variants in Sarcomeric Genes	1 (12.5)	6 (28.6)	5 (20.8)	0.524
NYHA Class				0.003
I	8 (100.0)	20 (95.2)	14 (58.3)	
II	0 (0.0)	1 (4.8)	10 (41.7)	
Abnormal ECG	6 (75.0)	16 (76.2)	23 (95.8)	0.225
MLVWT, mm	13.5 ± 5.6	15.5 ± 5.5	19.2 ± 5.9	0.080
MLVWT, z-score	9.9 ± 2.0	7.6 ± 3.6	10.1 ± 5.8	0.567
LVEF, %	62.8 ± 4.3	67.3 ± 4.2	66.9 ± 4.3	0.316
Follow-Up	9.8 ± 5.0	8.8 ± 4.8	9.7 ± 4.7	0.787
Hypokinetic End-Stage Evolution	0 (0.0)	6 (28.6)	7 (29.2)	0.216
MLVWT Thickening	0 (0.0)	8 (38.1)	8 (33.3)	0.123
MLVWT Thinning	2 (25.0)	1 (4.7)	0 (0.0)	0.029
No LV Remodelling	6 (75.0)	6 (28.6)	9 (37.5)	0.071

Abbreviations: ECG, electrocardiography; HCM, hypertrophic cardiomyopathy; LV, left ventricular; LVEF, left ventricular ejection fraction; MLVWT, maximal left ventricular wall thickness; NYHA, New York Heart Association; P/LP, pathogenic/likely pathogenic; SCD, sudden cardiac death.

**Table 4:** Baseline clinical characteristics of the study cohort according to left ventricular remodelling pattern during follow-up. Data are presented as mean ± SD, median (IQR), or *n* (%).

Clinical Features	No LV Remodelling (*n* = 21)	MLVWT Thickening (*n* = 16)	MLVWT Thinning (*n* = 3)	Hypokinetic End-Stage Evolution (*n* = 13)	*p*-Value
Age at Baseline, years	7.0 ± 5.7	10.2 ± 4.8	3.0 ± 5.2	11.3 ± 4.1	0.021
Males	11 (52.4)	15 (93.7)	2 (66.7)	8 (61.5)	0.058
Family History of HCM	10 (47.6)	9 (56.2)	1 (33.3)	6 (46.2)	0.876
Family History of SCD	8 (38.1)	3 (18.7)	1 (33.3)	6 (46.2)	0.445
Tested for Sarcomeric Variants	14 (66.7)	10 (62.5)	2 (66.7)	12 (92.3)	0.297
P/LP Variants in Sarcomeric Genes					
*MYBPC3*	3 (14.3)	2 (12.5)	1 (33.3)	0 (0.0)	0.214
*MYH7*	2 (9.5)	3 (18.7)	0 (0.0)	7 (53.8)	0.077
*TNNT2*	0 (0.0)	0 (0.0)	0 (0.0)	3 (23.1)	0.070
*TPM1*	1 (4.8)	1 (6.2)	0 (0.0)	0 (0.0)	0.723
Sarcomeric Negative	8 (38.1)	4 (25.0)	1 (33.3)	2 (15.4)	0.209
Double P/LP Variants in Sarcomeric Genes	1 (4.8)	3 (18.7)	1 (33.3)	7 (53.8)	0.156
NYHA Class					0.054
I	19 (90.5)	14 (87.5)	2 (66.7)	7 (53.8)	
II	2 (9.5)	2 (12.5)	1 (33.3)	6 (46.1)	
Abnormal ECG	19 (90.5)	13 (81.2)	2 (66.7)	11 (84.6)	0.489
MLVWT, mm	15.6 ± 5.9	14.7 ± 5.3	16.7 ± 6.4	21.6 ± 21.6	0.006
MLVWT, z-score	8.8 ± 4.4	7.1 ± 3.9	12.0 ± 2.6	11.2 ± 5.4	0.072
LVEF, %	64.4 ± 15.2	67.8 ± 4.4	65.7 ± 5.1	65.2 ± 4.2	0.815
Follow-Up	8.5 ± 5.0	10.5 ± 4.3	10.0 ± 4.3	9.3 ± 5.1	0.639

Abbreviations: ECG, electrocardiography; HCM, hypertrophic cardiomyopathy; LV, left ventricular; LVEF, left ventricular ejection fraction; MLVWT, maximal left ventricular wall thickness; NYHA, New York Heart Association; P/LP, pathogenic/likely pathogenic; SCD, sudden cardiac death.

**Table 5:** Univariate and multivariate analysis for hypokinetic end-stage evolution predictors at α level of 0.05.

Clinical Parameters	Univariate Analysis		Multivariate Analysis	
	OR (95%CI)	*p*-Value	OR (95%CI)	*p*-Value
Age, per 1 year	1.13 (0.99–1.30)	0.066	-	-
Male Sex	0.69 (0.19–2.53)	0.571	-	-
P/LP Variants in Sarcomeric Genes	5.00 (0.91–27.42)	0.064	-	-
P/LP Variants in *MYH7*	5.88 (1.30–26.51)	0.021	5.14 (0.92–28.59)	0.061
NYHA Class II	6.00 (1.42–25.27)	0.015	3.09 (0.55–17.36)	0.199
MLVWT, per 1 mm	1.22 (1.07–1.40)	0.003	1.17 (1.01–1.36)	0.043

Abbreviations: MLVWT, maximal left ventricular wall thickness; NYHA, New York Heart Association; P/LP, pathogenic/likely pathogenic.

## Data Availability

The data supporting the results of this study are available on request from the corresponding author.
